# Distribution and habitat attributes associated with the Himalayan red panda in the westernmost distribution range

**DOI:** 10.1002/ece3.7297

**Published:** 2021-04-02

**Authors:** Saroj Shrestha, Arjun Thapa, Damber Bista, Natasha Robinson, Ang Phuri Sherpa, Krishna Prasad Acharya, Shant Raj Jnawali, Sonam Tashi Lama, Sony Lama

**Affiliations:** ^1^ Red Panda Network Kathmandu Nepal; ^2^ Small Mammals Conservation and Research Foundation Lalitpur Nepal; ^3^ Wildlife Science Unit School of Agriculture and Food Sciences The University of Queensland Gatton Qld Australia; ^4^ National Environmental Science Program Threatened Species Recovery Hub Fenner School of Environment and Society The Australian National University Canberra ACT Australia; ^5^ Ministry of Forests and Environment Kathmandu Nepal; ^6^ WWF Nepal Kathmandu Nepal

**Keywords:** biological corridor, distribution, habitat requirements, red panda

## Abstract

The Himalayan red panda (*Ailurus fulgens*), a recently confirmed distinct species in the red panda genus, is distributed in Nepal, India, Bhutan, and south Tibet. Nepal represents the westernmost distribution of the Himalayan red panda. This study aims to determine important habitat features influencing the distribution of red panda and recommend possible habitat corridors. This manuscript described current potential habitat of 3,222 km^2^ with the relative abundance of 3.34 signs/km in Nepal. Aspect, canopy cover, bamboo cover, and distance to water were the important habitat attributes. It suggested five potential corridors in western Nepal. Overall, the study has important implications for conservation of the Himalayan red panda in western distribution range.

## Graphical abstract

Himalayan red pandas were confined between the range of 2,600 m–3,600 m, with most records between 3,250 m–3,400 m on the north and west‐facing slopes. The potentially suitable habitat of the red panda in Western Nepal is estimated to be about 3,222 km^2^ with a relative abundance of 3.34 signs/km.

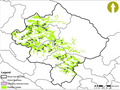

## 1 INTRODUCTION

Information on species distribution and habitat use is one of the important aspects of wildlife ecology. Such information is critical for the successful conservation of the species (Braun, 2005). Species distribution models are being widely used in wildlife studies to answer some of those key biological questions (Sinclair et al., 2006). Distribution models establish a quantitative relationship between the relative occurrence of species and their bio‐physical and environmental conditions in the landscape (Elith et al., 2006; Guisan & Zimmermann, 2000; Phillips et al., 2004, 2006; Phillips & Dudík, 2008). These models can provide essential information on habitat suitability, and key habitat attributes affecting species’ distribution. Such information obtained from the species distribution modeling eventually help identify and prioritize key conservation areas (Wilson et al., 2009).

Despite its broad geographical range across the Himalayas, Himalayan red panda (*Ailurus fulgens*) is patchily distributed and occurs at low densities (Thapa et al., 2018; Wei et al., 1999; Yonzon & Hunter, 1991a). The red panda has been recorded within the altitudinal range of 1,500–4,800 m (Choudhury, 2001), with a resemblance to that of habitats with dense, undergrowth bamboo (Choudhury, 2001, 2006; Zhang et al., 2006). Red pandas are charismatic animals that make them an ideal flagship species for harnessing public support for biodiversity conservation (Yonzon & Hunter, 1991a; Dorji et al., 2012). However, this endangered species is vulnerable to extinction due to habitat loss and fragmentation (Acharya et al., 2018; Glatston et al., 2015; Hu, 2001; Williams, 2003; Pradhan et al., 2001; Choudhury, 2001; Wei, Feng, Wang, & Hu, 1999; Glatston, 1994). The poaching and demand of hides have further aggravated the threat in Nepal than other range countries (Bista et al., 2020). A new study classified red panda into two distinct species: Himalayan red panda and Chinese red panda (*Ailurus styani*) (Hu et al., 2020). Of these, the former species inhabits in Nepal and is more vulnerable to threats compared to its Chinese relative (Hu et al., 2020).

Red panda is a habitat and diet specialist species. Available studies suggest that bamboo cover, tree canopy cover, and proximity to water are important attributes (Bista et al., 2019, 2017; Thapa et al., 2020). The major contribution of the red panda diet comprised of Bamboo (Dorji et al., 2011; Yonzon & Hunter, 1989) where leaves and shoots constitute 83% of the overall diet of red pandas (Yonzon & Hunter, 1991b).

Being primarily a bamboo eater, the red panda has a very low metabolic rate (Wei et al., 2000). Red panda spends most of the time on foraging and sleeping on tree branches or in tree hollows during the day (Wei & Zhang, 2011; Yonzon & Hunter, 1991a). This animal use elevated objects, such as shrub branches, fallen logs, or tree stumps to reach bamboo leaves. This elusive animal frequently uses specific latrine sites for defecation (Yonzon & Hunter, 1989). In Nepal, red panda presence has been documented from 24 districts and seven protected areas with potential habitat of 13,800 km^2^ to 24,000 km^2^ (Bista et al., 2017; Panthi et al., 2019; Thapa et al., 2020).

Similarly, occurring in a remote part of the Himalayan landscape, the red panda species remains poorly known, and the available database of the total population of species is possibly underestimated due to scarce records of occurrence. To date, most studies have focused on observational surveys of indirect indicators like feces and pugmarks (Bertolino et al., 2020; Pecchi et al., 2019; Pradhan et al., 2001; Wei, Feng, Wang, & Hu, 1999; Yonzon & Hunter, 1991a) as well as discussions with specialists and local communities (Jnawali et al., 2012). A comprehensive study on distribution and habitat attributes in the western range is still lacking. Potential distribution and habitat‐related attributes associated with red panda distribution have been broadly examined in Nepal (Bista et al., 2019, 2017; Thapa et al., 2020; Williams, 2003). However, very few studies have discussed on red panda distribution and habitat use in the western range (Bhatta et al., 2014; Bista et al., 2019). Available studies have either covered western range in a small‐scale (Bhatta et al., 2014) or covered it as a part of the large‐scale study (Bista et al., 2019; Panthi et al., 2019; Smeraldo et al., 2020). Long‐term conservation of red panda requires site‐specific detailed information from this range. In addition, the habitat use trend varies across the distribution range due to uneven micro‐habitat conditions (Bista et al., 2019). However, the available studies do not provide sufficient information on habitat use from the westernmost distribution range. These facts underpin the need of a comprehensive study in this one of the least explored red panda range.

With this background, our study aims to build understanding on distribution and habitat requirements of Himalayan red panda from its western distribution range. We intend to (a) document red panda distribution, (b) estimate abundance, and (c) identify possible habitat variables affecting distribution in the western Nepal. Understanding the impact of habitat use and distribution is important to conservation managers in planning effective conservation plans and mitigating the effect of development.

## 2 MATERIAL AND METHODS

### 2.1 Study area

**MAP 1:**
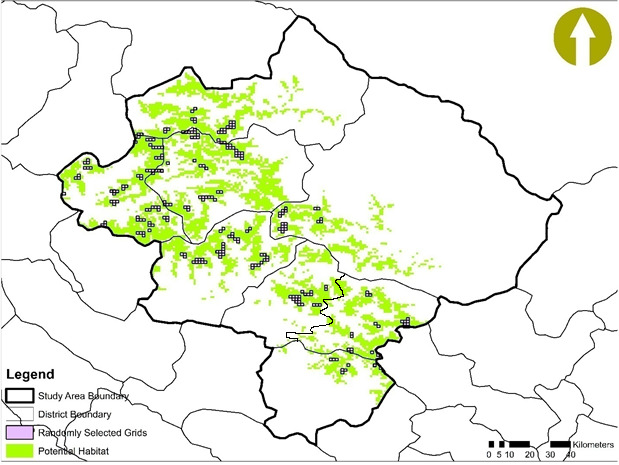
Study area

Geographically, Nepal is divided into three regions: Western (west to 83°E); Central (83°E to 86°30′E); and Eastern Nepal (east to 86°30′E) (Banerji, 1963; Stainton, 1972). Our study area included eight districts of Western Nepal: Kalikot, Mugu, Jumla, Jajarkot, Rolpa, Rukum‐East and Rukum‐West, and Dolpa (Map 1) and three protected areas, that is, Rara National Park, Dhorpatan Hunting Reserve, and Shey‐Phoksundo National Park in Western Nepal. The study area is located in western Nepal, covering an area of 22,682 km^2^. The western Nepal elevation ranges from 200 m to 8,091 m. The study area covers eight districts and three PAs. Red pandas have been documented in first two protected areas, that is, Rara National Park, Dhorpatan Hunting Reserve, and Shey‐Phoksundo National Park. The study area offers links between Rara National Park, Dhorpatan Hunting Reserve, and Shey‐Phoksundo National Park. Western Nepal most exhilarating and diverse topographies in Nepal (Acharya & Paudel, 2020).

The study area is home to diverse ecosystems. It has a large proportion of Nepal's birds (46%), mammals (42%), butterflies (22%), fishes (32%), reptiles (11%), amphibians (43%), and flowering plants (42%). Most of the flora and fauna are prevalent to Nepal and are globally threatened habitat loss, fragmentation, and climate change. Apart from these forests play an important role in sustaining livelihoods of the majority of population. The province is rich in aromatic and medicinal plants, in which Dolpa alone shares 57% (*n* = 400) of Nepal's medicinal and aromatic plants (Acharya & Paudel, 2020).

### 2.2 Data collection

The International Centre for Integrated Mountain Development's (ICIMOD) land use map (http://geoportal.icimod.org/) was used to identify forest cover area within elevation range: 2000 m to 4,000 m in potential red panda habitat. The DEM 90 m resolution image was used for elevation. The identified study area was divided into 504 grids of 9.6 km^2^ based on animal maximum home range (Yonzon & Hunter, 1989) using the Geospatial Modeling Environment built‐in ArcGIS 10.2 version.

**MAP 2:**
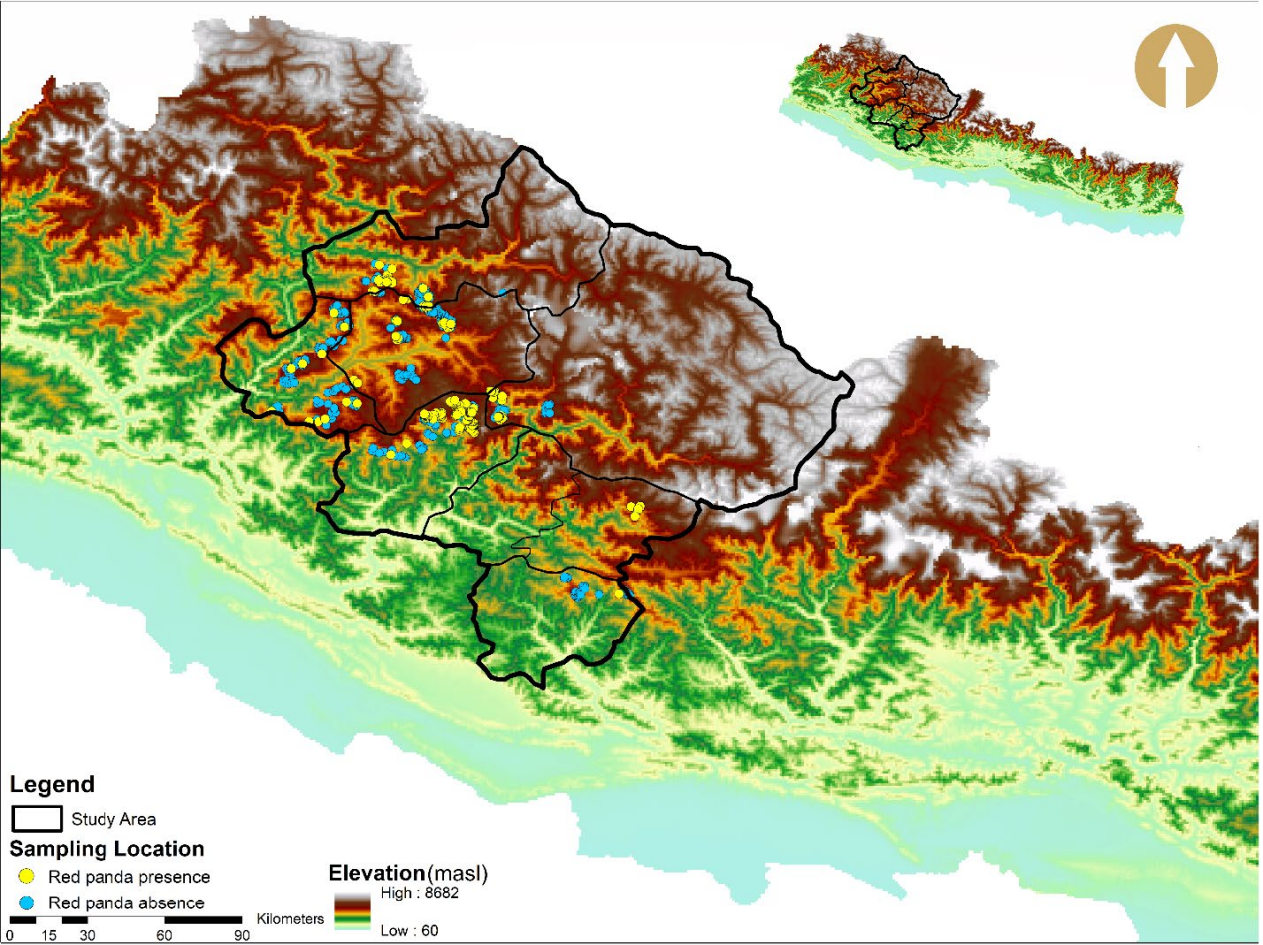
Study area with red panda presence and absence sign plots

We selected 50% of grids which were further divided into six sub‐grids (Area = 1.6 km^2^) to ease the data collection. Altogether, 252 sub‐grids were selected randomly for sampling across the habitat. We followed the red panda field survey and protocol for community base monitoring for data collection (MoFSC, 2015). Ensuring these selected sub‐grids cover the entire potential habitat, including elevation range and water availability, in the particular grid. All the available transects with an average length of 1 km at an interval of 100 m contour were traversed in each sub‐grid (MoFSC, 2015). However, it was not always feasible to conduct transect survey at an interval of 100 m mountainous topography. We recorded the red panda presence evidences based on indirect signs, such as droppings, foraging sign and remains of dead body parts, and direct sighting while walking along the transects. Additionally, we also recorded the occurrence data opportunistically when encountered beside designed transects. Additionally, the habitat variables were collected in a concentric sampling plots with a radius 10 m. Such sampling plots were also established in the red panda sign/sighting recorded site. Tree canopy cover and bamboo cover within a subplot of 10 m radius (A = 314.28 m^2^) and 1 m radius (3.14 m^2^) respectively also were recorded (MoFSC, 2015). In total, we covered 196 transects with 1,213 plots including 970 occurrence and 243 nonoccurrence plots along 100.68 km long transects. All the field survey was conducted in June–July and September–October (Map 2).

### 2.3 Data filtering and processing

Mostly, fecal pellets of red panda scat were used as indicative evidence of Himalayan red panda presence. The occurrence records (*n* = 331) were used to predict distribution of Himalayan red panda using species distribution modeling techniques. Also, we categorized other field‐collected data into three separate groups: topographic, habitat, and disturbance variables. All data were imported into excel spreadsheets and further statistical analysis was performed in R (Lê et al., 2008). Species absence record was nine times higher than the presence record, which could influence further statistical analysis. To address this inconsistency, we excluded those records consisting of more than 80% zero input values, and elevation below 2,000 m and above 4,000 m in further analysis.

Red pandas are relatively more abundant within these altitudinal range of (Choudhury, 2001; Pradhan et al., 2001; Yonzon & Hunter, 1991a). At the same time, we also removed all the outliers from the data.

### 2.4 Potential habitat and corridors

**FIGURE 1:**
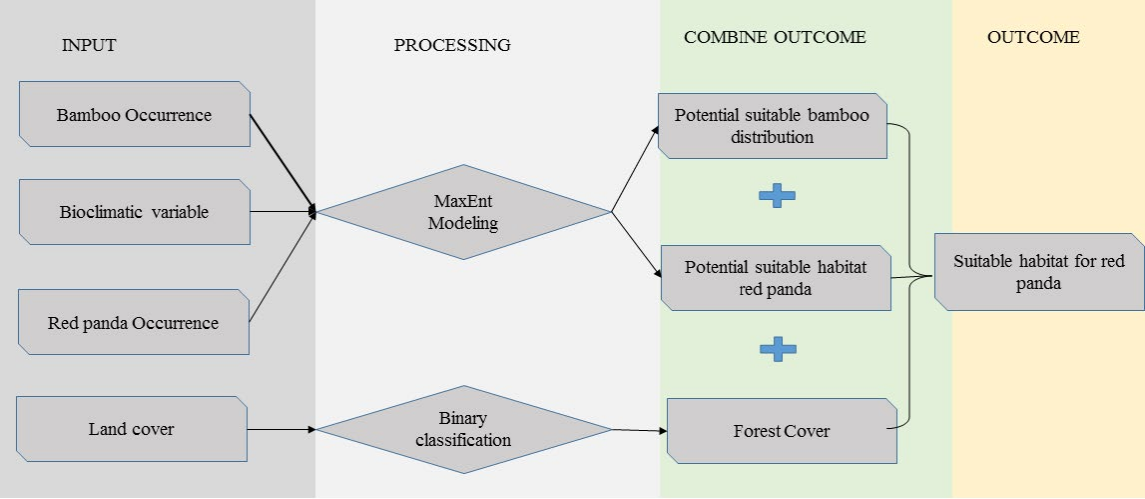
Modeling process for building potential habitat for the red panda

Occurrence data of red panda and bamboo species were extracted from the vegetation survey and used for distribution modeling based on the Maximum Entropy Algorithm (MaxEnt 3.3.3k). All 19 bioclimatic variables (11 temperature and 8 precipitation metrics) were downloaded from the WorldClim website (http://www.worldclim.org) (Hijmans et al. 2005). Our data were spatially distributed covering entire Western Nepal. All variables were converted into the ascii raster images with a cell size of 30 arc seconds (~1 km) and masked by study area boundary for the modeling process. We run 5,000 repetitions with a convergence threshold of 0.00001, a regularization multiplier of 1, a maximum number of 100,000 background points, the output grid format as “logistic,” algorithm parameters set to auto features, and all other parameters at their default settings. Random test percentage was 25% of presence locations to test the performance of the model. The model outcome was evaluated by the Area Under Curve (AUC) of the receiver operating characteristic (ROC) plot. The training and test AUC above 0.75 indicated a reasonable to high model discrimination ability and good model performance (Elith et al., 2006). The habitat suitability map was built by combining the habitat model, bamboo distribution model, and forest cover using raster calculator in ARC GIS 10.2. We reclassified the habitat into three suitability classes: low (0.10.50), moderate (0.50–0.75), and high (>0.75) (Shrestha & Bawa, 2014; Thapa et al. 2018). Forest and bamboo habitat within 2000 m and 4,000 m that consist river/stream, (sign recoded with 0.5), occurrence of low human footprint (https://sedac.ciesin.columbia.edu/data/set/wildareas‐v2‐human‐footprint‐geographic), and away from human settlements (excluded cattle sheds) (http://sedac.ciesin.columbia.edu/data/set/gpw‐v4‐population‐count‐rev11), and habitat patches (>9.6 km^2^) were considered as an area of potential corridor. All these layers were built and overlay using a spatial analysis tool in ArcGIS 10.2 (Figure 1).

### 2.5 Relative abundance and habitat association

Red pandas are elusive thus, it is hard to estimate their population size through direct sightings. So, the Encounter Rate (ER) of their signs was used as a standard method to measure their relative abundance (Bista et al., 2017; Ghose & Dutta, 2011). We calculated the ER as number of red panda signs per km. Habitat‐associated variables including topographic and vegetation covariates were analyzed using Principal Component Analysis (PCA) in FactoMineR package (Lê et al., 2008).

## 3 RESULTS

### 3.1 Sign abundance and potential distribution

**FIGURE 2:**
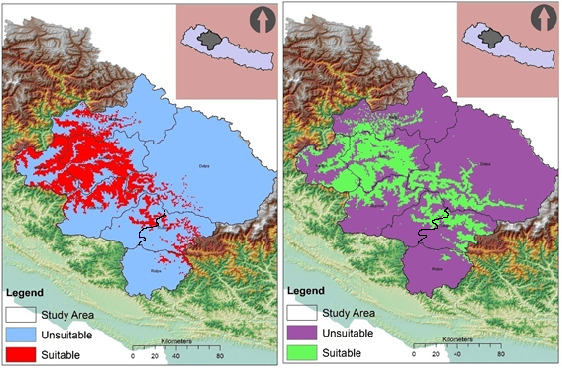
Potential suitable habitat for bamboo sp. (left red color indicates potential range of bamboo) and red panda (right: green color indicates potential ranges of red panda)

We recorded the presence evidence of red panda from 331 plots in the eight districts in the western Nepal (Table 1 and Figure 2). A total of 331 (98.51%) indirect signs and 5 (1.49%) direct signs were recorded. An average ER of 3.34 signs/km was recorded in the study area. The ER was significantly different in different districts. We recorded the highest ER in Jajarkot (6.23 signs/km) and least in Rolpa district (0.94 signs/km, Table 1).

**TABLE 1:** Relative abundance of red panda

Districts	No. of Sign	Transect surveyed (no.)	Length of transect (km)	ER (signs/km)
Jajarkot	181	48	29.00	6.23
Dolpa	37	26	8.54	4.33
Rukum‐East	14	17	3.06	4.75
Rukum‐West	10	12	3.00	3.33
Mugu	43	36	20.05	2.14
Kalikot	11	18	5.19	2.10
Jumla	32	27	23.36	1.36
Rolpa	8	12	8.48	0.94
Total	336	196	100.68	3.34

The training AUC and test AUC of the distribution model of red panda were 0.96 and 0.94 respectively. Similarly, the training AUC and test AUC of the potential bamboo species were 0.93 and 0.92 respectively (Figure 2). Both training and test AUC were higher than the random that indicated the model performed better than random. In western Nepal, potential habitat of red panda and bamboo distribution area was estimated to be 7,191 km^2^ and 6,294 km^2^ respectively. At total of 3,222 km^2^ area was estimated as the potential habitat when combing overlapping area among distribution of Himalayan red panda, bamboo species, and forest cover. (Table 2). The distribution model predicted more than two third of the total habitat available in only three districts, namely Jajarkot, Jumla, and Kalikot, while the remaining five districts constituted less than one third of the habitat (Table 2).

**TABLE 2:** Potential suitable habitat for red panda based on predicted red panda habitat, bamboo distribution, and forest cover

District	Area (km^2^)	Habitat (%)
Jumla	1,087	33.74
Kalikot	711	22.07
Jajarkot	516	16.01
Rukum‐East	220	6.82
Rukum‐West	120	3.72
Dolpa	251	7.79
Mugu	248	7.70
Rolpa	69	2.14
Total	3,222	100.00

**FIGURE 3:**
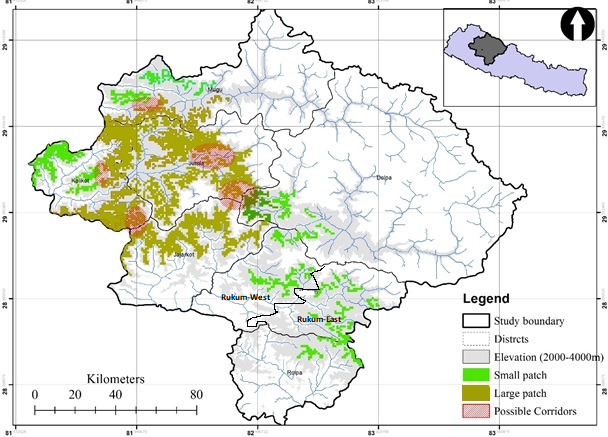
Suitable habitat patches and possible corridors

This predicted habitat was found to be fragmented into numerous patches. The four districts: Jajarkot, Jumla, Mugu, and Dolpa seemed to have conspicuous fragmentation than other four districts located in the east, which indicates the increasing fragmentation trend toward the west. (Figure 3).

### 3.2 Habitat association

**FIGURE 4:**
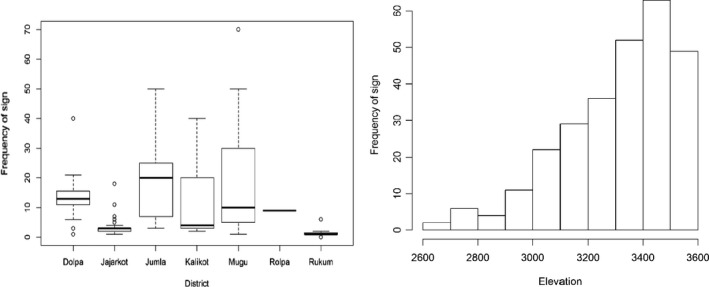
Distribution of sign records in different districts (left); record of sign along elevation in all study sites (right)

**FIGURE 5:**
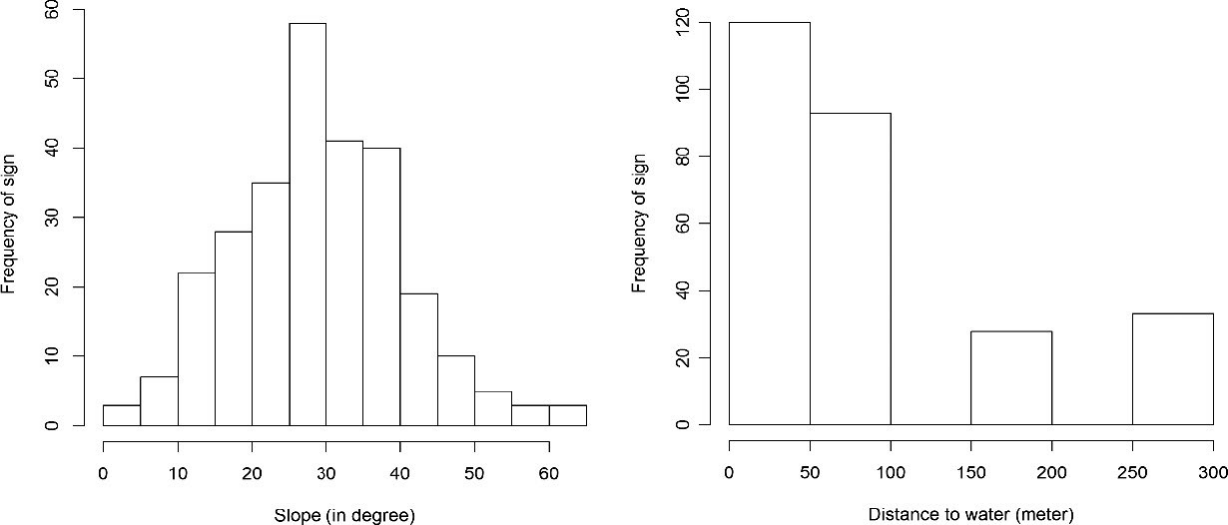
Records of red panda sign based on the slope (right); records of red panda sign‐based distance to water

We recorded the presence evidences between the elevation range of 2,600 m and 3,600 m (Figure 5). Out of those, 75% signs were recorded between 3,250 m and 3,400 m. Likewise, we recorded most of these signs in North‐West aspect (23.6%) followed by West (19.1%), North‐East (15.8%), and East (14.5%) aspects (Figure 4). Their least preference was observed in Southern aspects (9.5%, *p* = 6.29E−17). We recorded 90% of these presence signs within 150m of a water distance (overall mean ± *SD* =  101.32 ± 43.16 m, Figure 4). Similarly, we observed a large proportion (58%) of sign within the slope of 31.14°±5.18° (Figure 4). Altogether 278 fecal piles were observed on tree branches, fallen logs, ground, and rock. Trees were the most common substrate used for defecation (62.21%) followed by the ground surface (29.96%) and fallen logs (8.70%) and least in the rocks .

**FIGURE 6:**
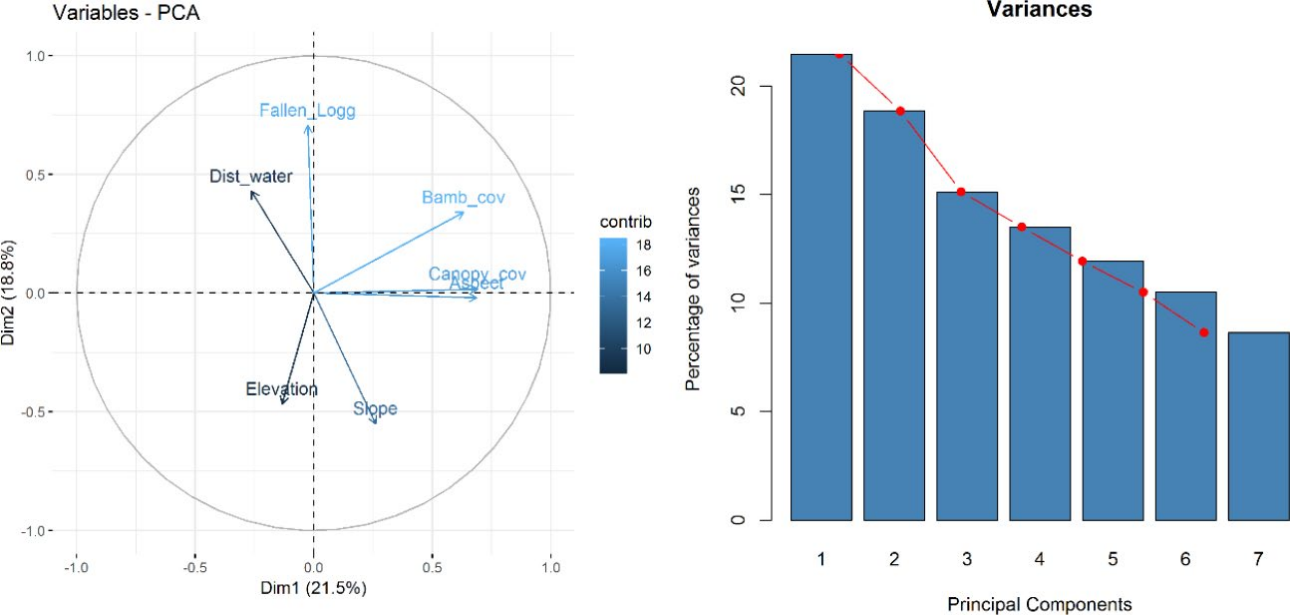
Biplot of principal component analysis (PCA) showing the relationship of habitat variables (right); a scree plot consisting a graph of the eigenvalues/variances associated with components (left)

**FIGURE 7:**
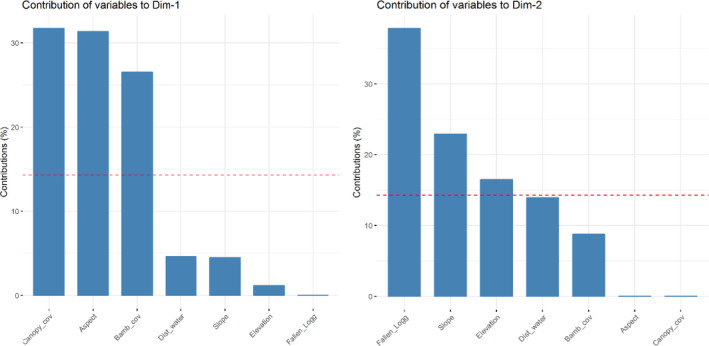
Contribution of variables to Dim.1 and Dim. 2 (left and right)

The highest eigenvalue obtained showed that seven dimensions were required to capture all the data variations. By visualizing the scree plot and variance, at least five dimensions were required to retain 80% of the variance in the data (Figure 6). The first two dimensions of this space were plotted to examine the association among the variables in the red panda habitat. Dimension 1 accounted for 21.5% of the variance in the data and dimension 2 accounted for 18.8% of the variance (Figure 7). Canopy cover, aspect, and bamboo cover explained over 60% with other variables having low contribution in the first dimension. Similarly, the fallen log, slope, and elevation had high contribution in the second dimension. The analysis showed that these are the key micro‐habitat variables influencing the distribution of red panda (Table 3).

**TABLE 3:** Principle component scores for PCA of habitat variables

Variables	Dim.1	Dim.2	Dim.3
Elevation	−0.133	−0.466	0.606
Aspect	0.687	−0.02	0.25
Slope	0.261	−0.55	−0.516
Canopy_cov	0.691	0.017	−0.352
Bamb_cov	0.632	0.34	0.303
Dist_water	−0.263	0.429	−0.372
Fallen_Log	−0.026	0.706	0.094

## 4 DISCUSSION AND CONCLUSION

The conservation status of the red panda is a barometer of regional conservation efforts for monitoring the integrity of the Eastern Himalayan Broadleaf and Conifer Eco‐region (Williams, 2003). This study represents a major advance on past sparse and the restricted study which has principally relied on historical anecdotal information. Using extensive field‐based survey ever conducted in Western Nepal, spanning red pandas’ range districts of Western Nepal, we demonstrate how bioclimatic variables can be used to identify potential suitable habitat along with primary habitat requirements, distribution, and potential corridor for the red panda conservation.

Based on extensive field survey and predictive potential habitat suitability model using bioclimatic variables to build a Maxent habitat model of red panda in Western Nepal. Our major findings have been (1) potentially suitable habitat in Westernmost range, (2) important attributes that influence red panda presence, and (3) number of potential corridors in Western Nepal. Our result with regard to total potential red panda habitat, habitat preference, and potential corridors in Western Nepal could be critical for setting appropriate management goals, monitoring effectiveness, informing policymakers, and other relevant stakeholders. Western Nepal harbors 3,222 km^2^, that is, 23.38% according to Panthi et al. (2019) and 14.86% as per Thapa et al. (2020) of potential red panda habitat in Nepal. This might suggest that this westernmost range has suitable ecological variables such as bamboo cover, canopy cover, aspects, and water body needed for red panda survival.

Our study concluded that tree and bamboo cover, proximity to the water body, and aspect were the strongest predictor of red panda distribution which is supported by findings of previous studies (Bista et al., 2017; Dorji et al., 2011; Pradhan et al., 2001; Thapa et al., 2018, 2020; Williams, 2006; Yonzon & Hunter, 1991a). Tree canopy cover has provided an ideal microclimatic setting (e.g., temperature and relative humidity; Anhuf & Rollenbeck, 2001; Thapa et al., 2018) which favored the better growth of the understory of bamboo (Thapa et al., 2018) and also provide shelter and protection (Bista et al., 2019; Pradhan et al., 2001; Thapa et al., 2020; Yonzon & Hunter, 1991a). Also, canopy cover contributes for the safety shelter away from the avian predators which could show the high record of fecal pellets in the tree branches. Also, 62.21% of the red panda latrine site was recorded on tree branches. This might be due to as tree provides better shelter and safety from predators and easy movement from the branches of trees (Pradhan et al., 2001; Reid et al., 1991; Thapa et al., 2020). For successful habitat management and species recovery, understanding habitat selection is critical (Yangb et al., 2020).

The presence of bamboos was observed in 85% of sign plots. This demonstrates the importance of bamboos as one of the fundamental parameters affecting red panda distribution (Dorji et al., 2011; Fox et al., 1996; Williams, 2004; Yonzon & Hunter, 1991b). This finding is similar to the findings of Thapa et al. (2020), Panthi et al. (2019), Thapa et al. (2018), Sharma et al. (2014), Pradhan et al. (2001) and Yonzon & Hunter (1989). Besides bamboo species, other food such as seasonal fruiting berries contribute a very little proportion in food of the red pandas (Yonzon & Hunter, 1991b). Such a near inclusive reliance on bamboo may be a survival strategy. Proximity to bamboo lessens the travel needed to forage, which conserve red panda energy (Hu, 2001; Reid et al., 1991).

Occurrence of Himalayan red panda is positively associated with distance to water resources. Almost 90% of red panda fecal pellets were recorded within 150 m which might suggest that red panda frequently need water for their physiological food processing due to poor digestive system. Our findings are identical to the results of previous studies (Bista et al., 2017; Dorji et al., 2011; Pradhan et al., 2001; Thapa et al., 2018; Williams, 2006; Yonzon & Hunter, 1991b). Distance to water is also important factor for giant panda for determining the habitat selection (Ye et al., 2007). A similar conclusion was drawn in a comparison of 18 giant panda in the Foping Nature Reserve, China. Giant panda here chose habitat close to water source (Deng, 1992). In Wolong reserve, Sichuan, China similar result was documented with Chinese panda (Reid & Jinchu, 1991). Such proximity might help the red panda to avoid predators such as snow leopard, marten, and human and conserve its energy (Bista et al., 2017; Pradhan et al., 2001). Proximity to the water is important to conserve their energy as they do not require a long distance to travel for water (Bista et al., 2017; Pradhan et al., 2001). Also, this might be also due to low water content associated with bamboo leaves (Reid et al., 1991; Wei et al., 1999). In Wolong Reserve, the water content in their scat was 72.4 ± 5.5%, and that in *B*. *faberi leaves* 59.7 ± 8.1% or less than 12.7% less than in the scat (Johnson et al., 1988). In the wild, their traces, including fecal pellets, foraging sites were frequently found at a site close to the water body. For instance, in Mabian Reserve, red pandas often foraged at sites less than 200 m away from the water body (Wei, Feng, Wang, & Hu, 1999). In CHAL, observation indicated that proximity to water may be an important habitat requirement because 90% of the fecal pellets were found within 100 m of the nearest water body (Bista et al., 2017).

Red pandas were detected more frequently on northerly and westerly slopes in our study, which supports similar observations by Yonzon & Hunter (1991a) in Langtang National Park. Yonzon et al. (1991a) hypothesized that the Northern slope receives fewer sunlight periods which are conducive to the growth of *fir‐jhapra* bamboo forest. In Jigme Dorji and Thrumshingla National Parks, Bhutan, red pandas were associated strongly associated with southerly slopes as southerly slope receives direct sunlight during winter and red pandas rested in direct sunlight during winter to reduce heat loss (Reid et al., 1991). We noted that northerly and westerly slopes in our study areas had relatively high bamboo densities, which might be associated with sunlight and rainfall. Due to the lack of detailed knowledge of bamboo ecology in Nepal, more studies are needed to investigate the effects of the physical landscape variables on bamboo species.

In Nepal, forests are integral to human subsistence, however, because of the common reliance on forests by humans and red pandas, sustainable management of natural resources is critical to meet the needs of both people and red panda conservation. This proposes that red pandas are primarily dependent on bamboo species. Bamboo species are vulnerable to climate change because of their uncommon reproduction intervals (Janzen, 1976), apart from limited seed dispersal capacity (Taylor et al., 1991; Tuanmu et al., 2013). To ensure red panda survival bamboo conservation should be the highest importance. Therefore, the establishment of red panda focused conservation zones is needed to secure the long‐term survival of red pandas through maintaining habitat connectivity that ensures the conservation of a genetically viable population in the long run. This study recommended potential five potential corridors in western Nepal. Although a detailed field‐based assessment is needed to validate it scientifically.

The present study identified five potential corridors in Western Nepal with an important cluster in Jumla, Jajarkot, and Dolpa district. These corridors are the vital repository of biodiversity in this region because they connect Nepal's largest protected area, that is, Shey‐Phoksundo National Park with Rara National Park and Dhorpatan Hunting Reserve. Although biodiversity of the suggested corridor has not been affected by any detrimental development activities, the same cannot be forecast for the future. It is imperative that the partnerships and collaborations with the community should be nurtured to ensure the long‐term conservation of suggested the corridor. This study also further highlighted the need for site‐specific conservation strategies that need to be bolstered with comprehensive information on bamboo distribution, other palatable species, and microclimatic conditions.

## CONFLICT OF INTEREST

The authors have declared that no competing interests exist.

## AUTHOR CONTRIBUTION

**Saroj Shrestha:** Conceptualization (lead); Data curation (lead); Investigation (lead); Project administration (lead); Supervision (lead); Writing‐original draft (lead). **Arjun Thapa:** Data curation (equal); Methodology (equal); Writing‐review & editing (equal). **Damber Bista:** Supervision (equal); Writing‐review & editing (equal). **Natasha Robinson:** Writing‐review & editing (equal). **Ang Phuri Sherpa:** Funding acquisition (lead); Project administration (lead). **Krishna Prasad Acharya:** Resources (equal); Writing‐review & editing (equal). **Shant Raj Jnawali:** Funding acquisition (equal); Resources (equal); Writing‐review & editing (equal). **Sonam Tashi Lama:** Investigation (equal); Project administration (equal). **Sony Lama:** Data curation (equal); Writing‐review & editing (equal).

## Data Availability

https://doi.org/10.5061/dryad.dv41ns1x7.
